# A new method to produce T-shaped tubular fittings with experimental and simulation results

**DOI:** 10.1371/journal.pone.0214608

**Published:** 2019-04-08

**Authors:** Atef M. Ghaleb, Mohamed A. Saleh, Adham E. Ragab, Abdulmajeed Dabwan, Tamer M. Khalaf, Usama Umer

**Affiliations:** 1 King Saud University, Industrial Engineering Department, King Saud University, Riyadh, Saudi Arabia; 2 Private consultant, Cairo, Egypt; 3 Department of Mechanical Engineering, College of Engineering, Al-Azhar University, Cairo, Egypt; 4 Advance Manufacturing Institute, College of Engineering, Saudi Arabia; Jamia Millia Islamia A Central University, INDIA

## Abstract

A tube is an important structural element for fluid manipulation in piped networks in many industries. Tube branching is achieved using tube fittings of various shapes, including T, Y, X, and L shapes. This study proposes a new innovative technique to produce T-shaped tubular fittings. The technique uses a specially designed die setup where a tube is placed inside a T-shaped die cavity and a metallic insert is used to deform the tube into the cavity, creating the T-fitting shape. Experimental and numerical methods are used to evaluate the process. The main outcome of this research is the successful creation of T-shaped copper tube fittings using a technique similar to tube hydroforming without the need for internal pressure. This technique could be modified to assist the production of T-fittings with thicknesses outside the hydroforming limits.

## Introduction

Tubes are cylindrical structural elements typically used for retaining and manipulating fluids. The direction of fluid flow can be manipulated through tube branching that uses tube fittings of different shapes, for example, T, Y, or X shapes [1,2]. T-shaped tubular fittings include two branches, or arms, with three ends and are capable of directing the fluid into two different directions.

Recently, there has been an increasing demand for tubes that have the T-shape configuration, particularly, in automotive industry, aerospace industry, and for household appliances [3–5]. Various methods have been used for forming T-shaped tubular fittings with different tube sizes: diameters, wall thicknesses, and lengths.

Welding, casting, and hydroforming are typical methods that are currently employed to produce T-shaped tubular fittings. Tube hydroforming (THF) involves the use of controlled internal pressure to expand a tube into a T-shaped part. Despite being one of the most widely used methods [6, 7], the THF process has some drawbacks, such as long cycle time, expensive specialized equipment [8–11], and limited applicability in high pressure environments due to fluid leakage, as well as in high temperature environments due to low fluid evaporation and ignition temperatures.

To improve tube formability in THF processes, researchers applied several techniques to reduce thinning of the tube wall and to increase the possible height of the emerging branch. Huang et al. [12] used the adaptive support vector regression during the T-shaped THF process to optimize the loading path. In a different research, Hwang et al. [13] suggested a control algorithm to identify suitable loading paths in the T-shaped THF process with various outlet diameters. A sensible loading path was modified to produce the part successfully.

Kadkhodayan et al. [14] examined Y- and X-shaped THF using numerical simulation and used simulated annealing algorithms to optimize the loading path. With the aid of the proposed method, the tube was formed with better formability indicators and lower capacity machines. Brooghani et al. [15] used the multilevel response surface method with numerical simulation during the production of T-shaped tubes to optimize the loading path. They concluded that multilevel RSM is effective in improving the loading path to produce more uniform thickness.

Furthermore, the friction between the tube and dies during THF process is an important parameter that affects the tube formability due to the phenomena of metal flow repression. Guo et al. [16] examined the influence of lubrication on the height of protrusion and tube thickness of a “316 L SS/All clad” T-branch. They concluded that the height of protrusion increased and the thinning decreased at low friction coefficients. Similar results were reported by Ahmadi et al [17].

In addition, Cheng et al. [18] conducted simulation and experiments for hydroforming Y-shape tubes using annealed copper tubes. They found that the minimum wall thickness was at the top of a protrusion with maximum thinning of 33%. These results matched those of Mingtao et al. [19], who, using a special 3-stage punch shape, revealed that the maximum thinning for copper T-shaped tubes reached 31.3%. Mingtao et al. also found that the maximum effective strain and stress were 0.65 and 487.8 MPa, respectively. Maximum stress of T-shape hydroformed tubes was also examined by Crapps et al. [20] who estimated it as 475 MPa using finite element simulation.

Thus, a system for forming T-shaped tubular fittings that solves the aforementioned problems is desired. The objective of this research is to develop an innovative technique to produce T-shaped tube fittings. The suggested technique resembles the hydroforming process but without the need for internal pressure.

## Details of suggested technique

**Fig 1:**
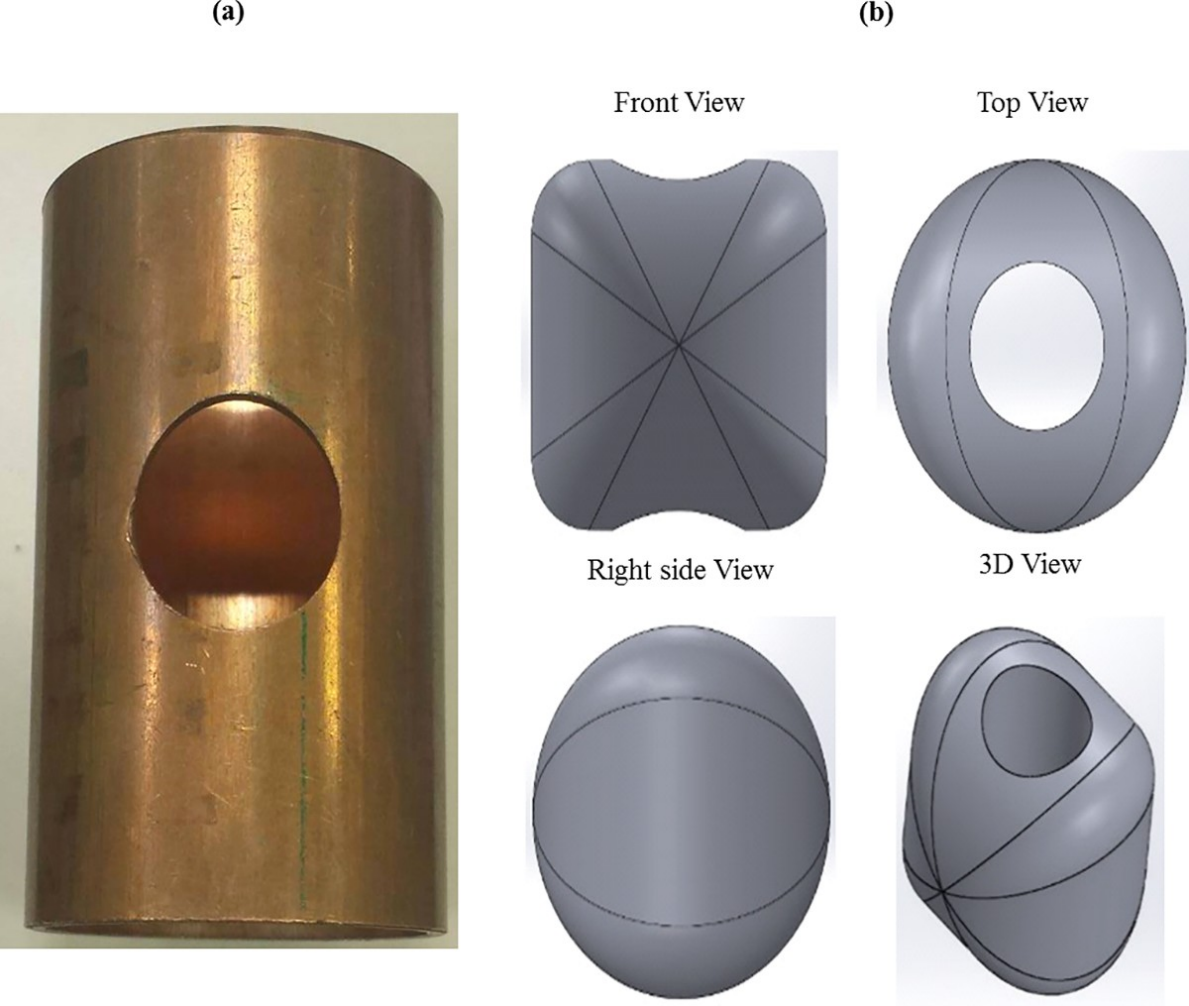
(a) the tube workpiece (b) the insert.

**Fig 2:**
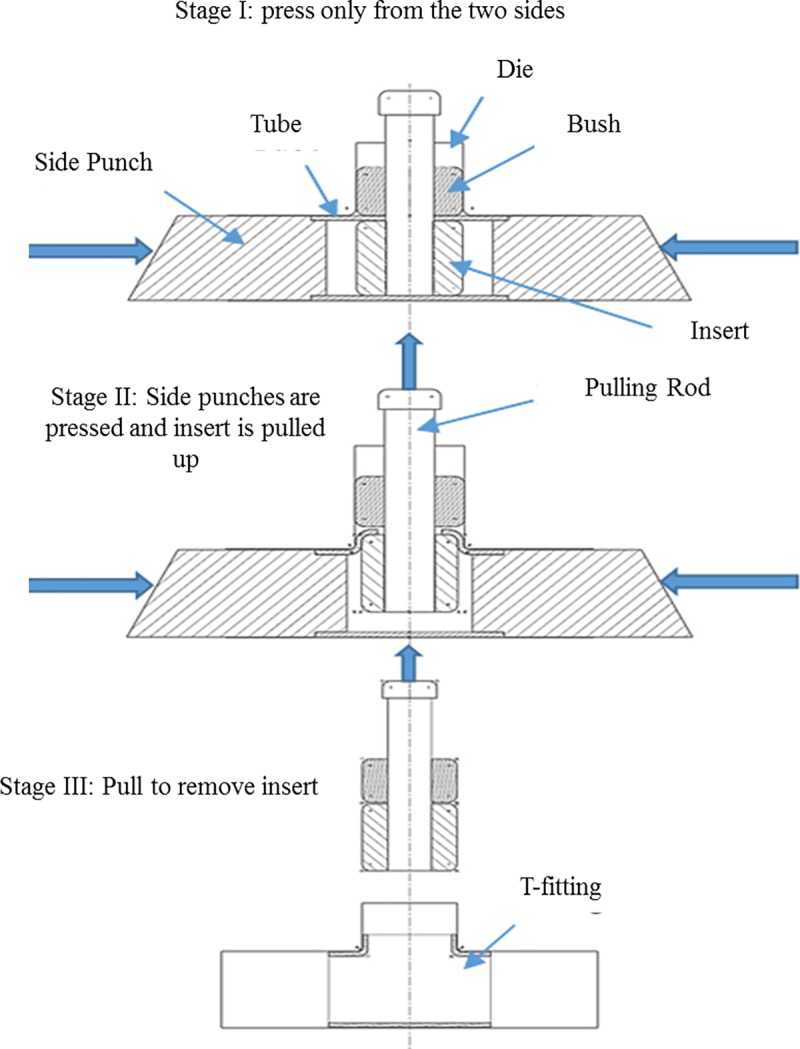
Production of a T-tube fitting.

The proposed forming process produces a T-shaped tubular part from a straight tube. In this process, the tube to be formed is placed inside a die that has an internal T-shaped cavity. The tube workpiece has to be holed in the middle prior to this step, as shown in Fig 1A. An insert consisting of a metallic part with the geometry shown in Fig 1B is inserted into the tube and fastened to a rod that passes through the hole in the middle of the tube. The insert is pulled by the rod and, simultaneously, two punches apply pressure on the tube sides. The insert pulls the tube into the T-shaped cavity, thereby producing the required shape. These steps are illustrated in Fig 2.

**Fig 3:**
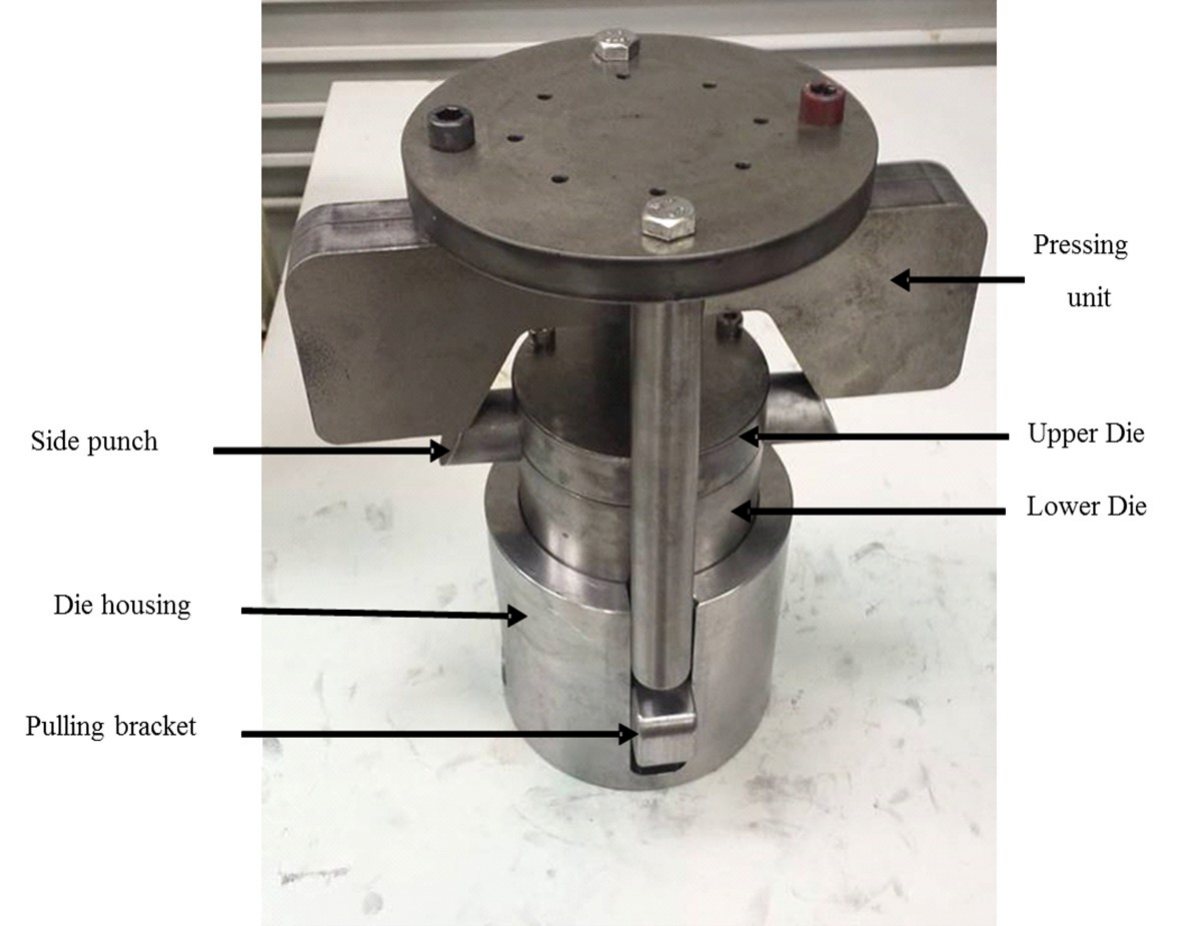
Die set assembly.

**Fig 4:**
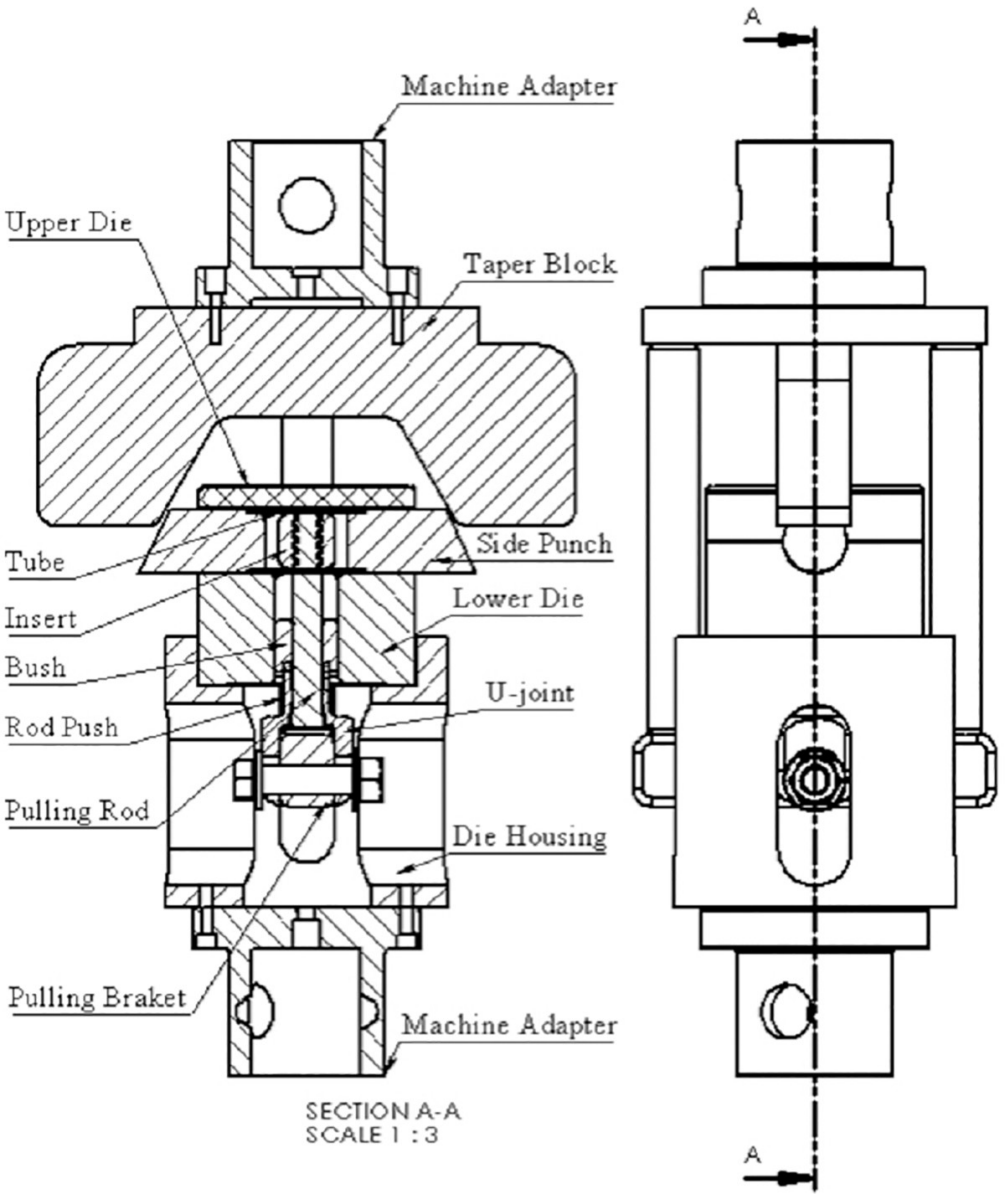
Die set detailed assembly drawing.

A special die set was designed and manufactured to produce the T-shape tubes. The die set assembly is shown in Fig 3. The main components of the die set are: upper die, lower die, pulling rod, insert, pulling bracket, bush, die housing, pressing unit, side punch, and U-joint (the interior parts of the die are not shown in Fig 3). A detailed assembly drawing of the die set is shown in Fig 4. All the die components are made from heat-treated D2 tool steel. The die was split into two parts for easy removal of the product. The insert was designed to geometrically resemble the intersection of two perpendicular tubes with a diameter equal to the inner diameter of the worked tube. The aim of this particular geometry is to facilitate the forming process with minimum stresses on the tube to reduce the chances of failure.

## Methodology

### Material

**Fig 5:**
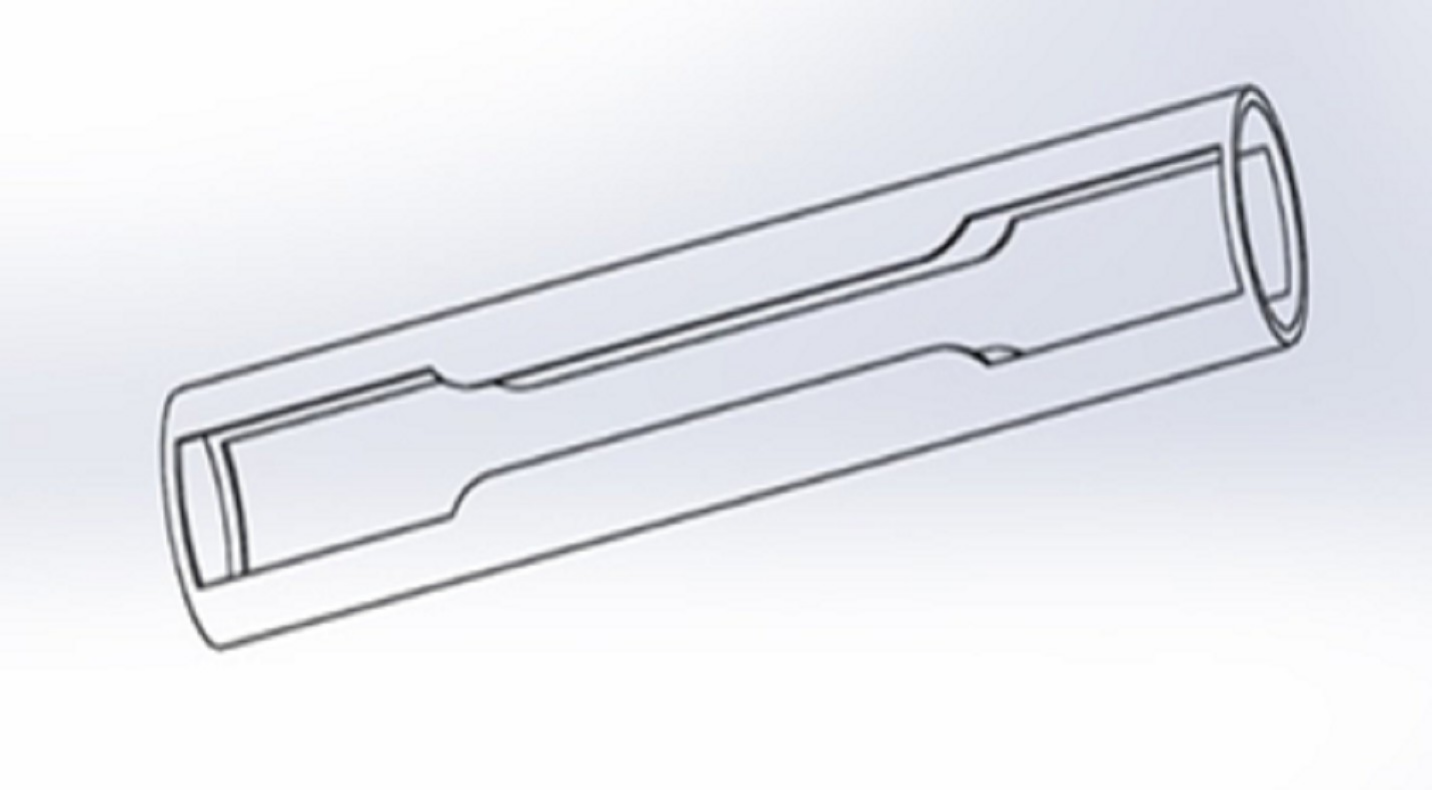
Tube after cutting the longitudinal tensile test specimens.

In this study, the produced T-fitting is made of pure annealed copper with a thickness of 1.6 mm. Tensile test specimens were prepared according to ASTM standard E8-04 (cut from the tube by Electrical Discharge Machining (EDM) and flattened by plastic hammer) to be tested on an Instron Tensile Testing Machine. Fig 5 shows the tube after cutting the test specimens and the materials properties obtained from uniaxial tensile testing (using strain rate 10^4^ s^-1^) are shown in Table 1.

**Table 1:** Measured mechanical properties of annealed copper.

Young's modulus [MPa]	Poisson ratio	Strength Coefficient (k)	Hardening exponent (n)	Ultimate tensile strength [MPa]	Yield stress [MPa]
112000	0.28	608	0.644	223.7	60

### Experimental method and procedure

#### Specimen preparation

**Fig 6:**
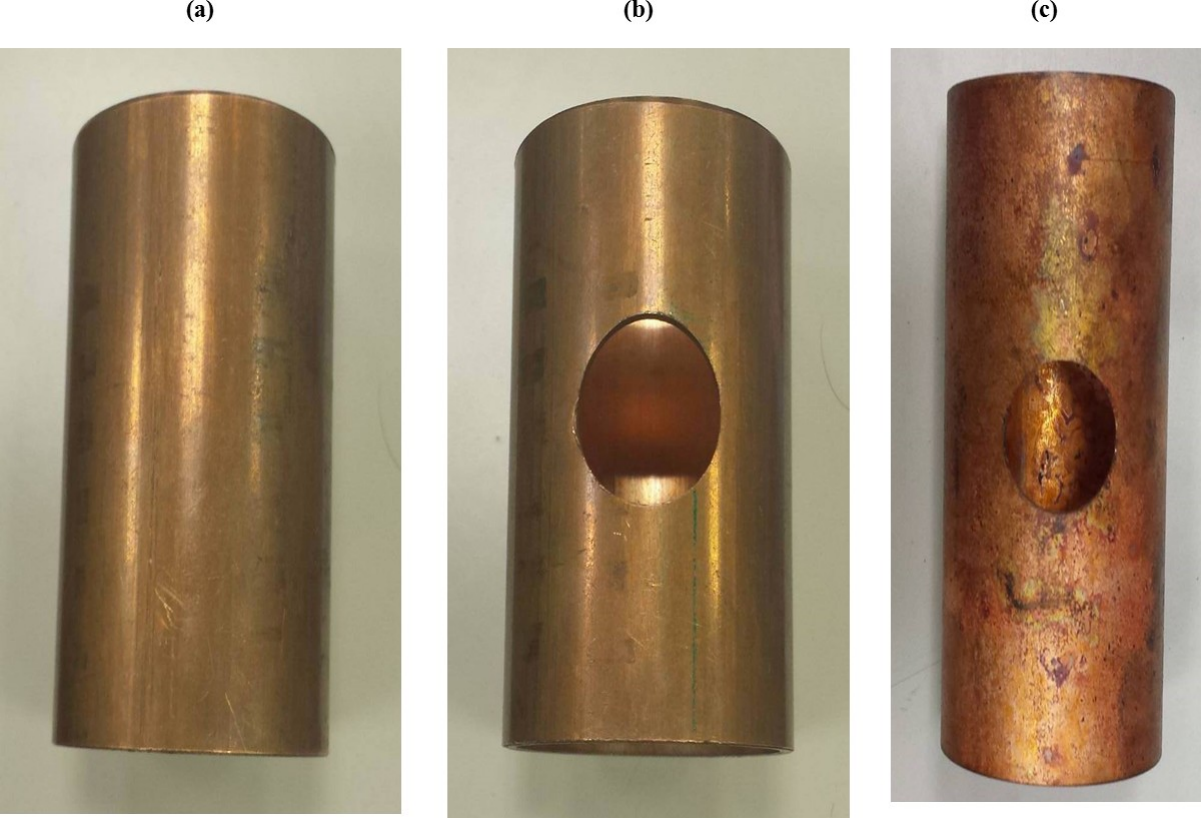
Specimen preparation: (a) Cut (b) Drilled (c) Annealed.

Using a lathe, a workpiece of the appropriate length was cut from a copper tube with 35mm outer and 31.8mm inner diameter. Holes were then drilled in the middle of the workpiece using a drilling machine. Different hole diameters (16, 18, 20, and 22mm) were tested to produce the fittings without defects, and a hole of diameter 16mm was the most suitable choice for the 35mm tube. The samples were annealed using a Nabertherm P330 oven at 550°C for 30 minutes [21] then quenched in water at room temperature to improve their formability. Fig 6 shows the specimen shapes at different procedure steps.

Three stages of operation were carried out during the forming process, namely stages I, II, and III, as shown in Fig 2. In stage I, the right and left punch pressed the tube from both sides using appropriate linear displacement. The purpose of this stage is to avoid expected wrinkling by applying pre-straining to the workpiece. In stage II, the insert was pulled through the hole while the side punches pressed the tube from both sides. In stage III the side punches are stopped, and the vertical displacement continues until the insert is removed.

#### Lubricant

Lubrication plays an important role in the process as it reduces friction between the tool-workpiece and die-workpiece interfaces. Castrol oil mixed with fine graphite silver powder was used as a lubricant in the current experiments.

## Design of experiment (DOE)

**Fig 7:**
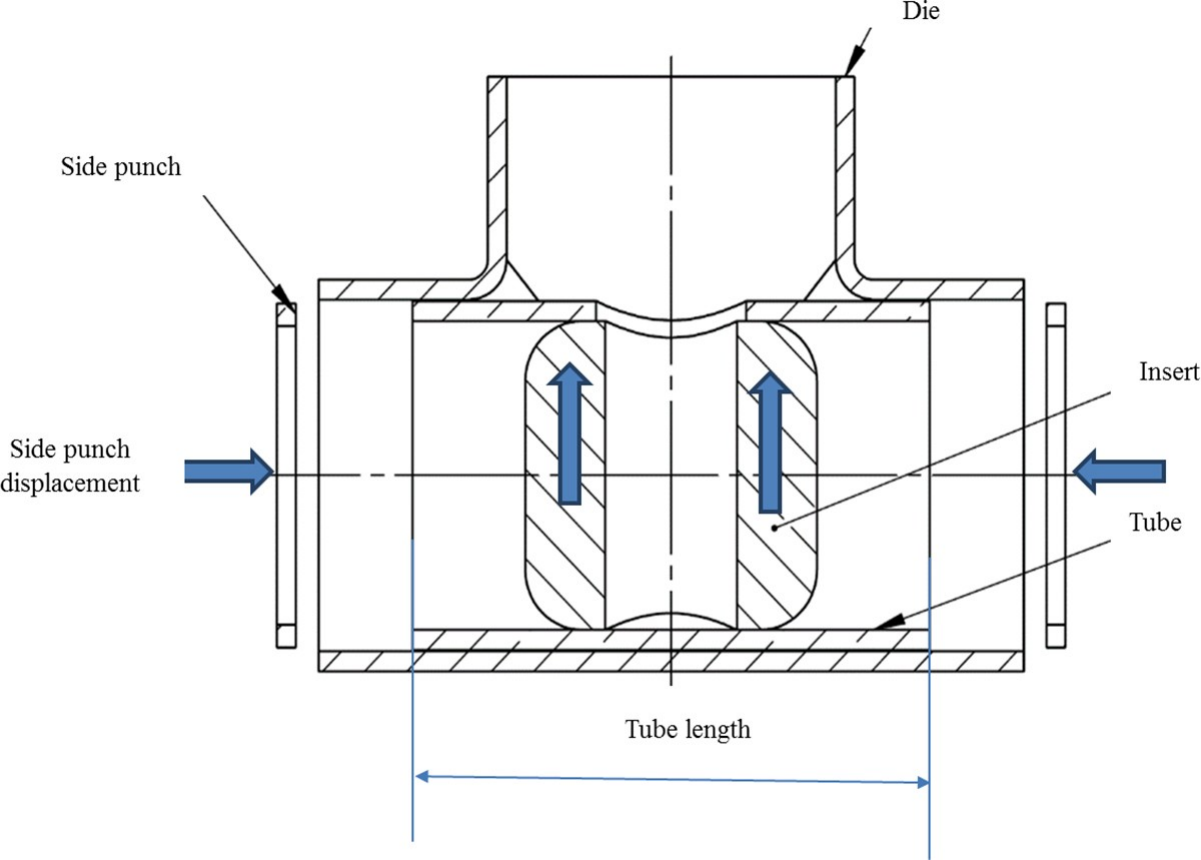
T-shaped tube fitting process parameters.

**Fig 8:**
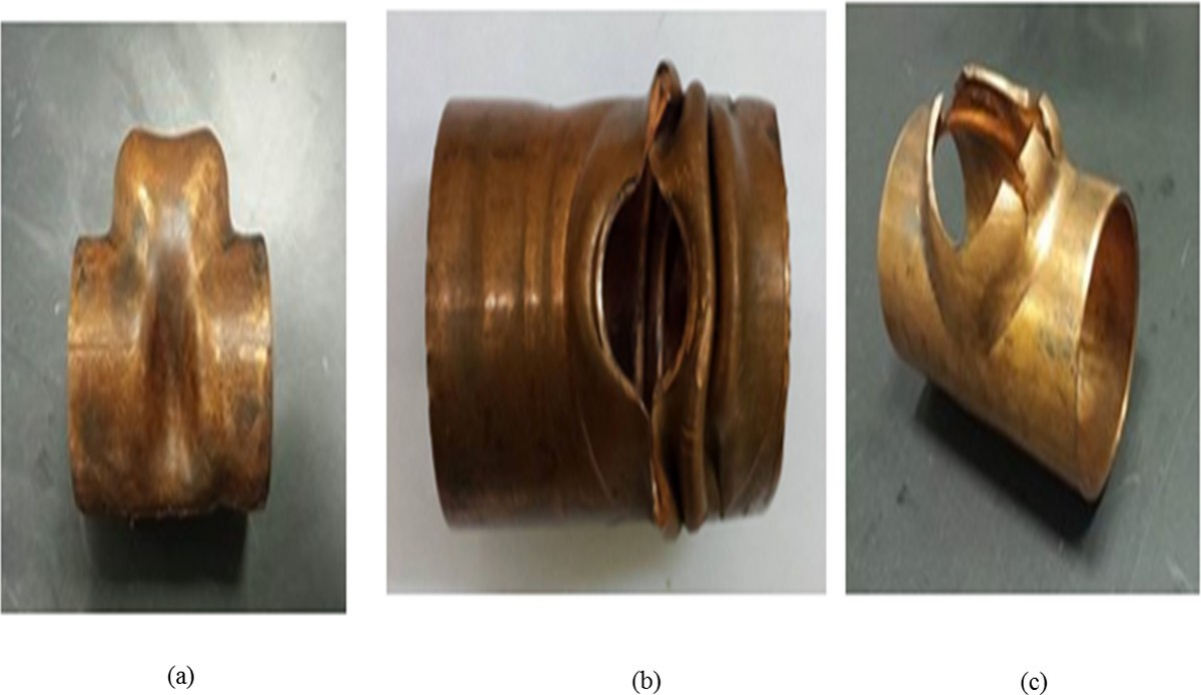
Process failure samples: a) Wrinkling b) Buckling c) Bursting.

DOE is an efficient technique to analyze the effects of different variables and interactions on measurable outputs. The output under consideration in this study was the tube thinning as it affects the functionality and safety of the tube products. The influence of two process parameters, namely, (1) tube length and (2) side punch initial displacement on the tube thinning was investigated. The two parameters are illustrated in Fig 7. To determine parameter levels, screening experiments were conducted until a failure occurred, as shown in Fig 8. In part *a)* of Fig 8, the wrinkling occurs due to less side punch displacement is necessary, while in *b)* buckling occurs due to more side punch displacement is required. The bursting in *c)* happened to the tube that had not been annealed.

The experimental parameters level values presented in Table 2 were selected to avoid tube failure. Table 3 summarizes the process parameters for the nine experiments (Cases 1–9). Each experiment was replicated three times totaling 27 runs. It is worth noting that the use of the selected parameters’ values is limited to tubes of the same material and dimensions. Different tubes will require different values to produce a sound product.

**Table 2:** Design of experiment (DOE) factor levels.

Experimental parameters	Low level	Medium level	High level
Tube length (mm) (TL)	60	65	70
Side punch displacement (mm) (SPD)	6	7	8

**Table 3:** Experiment runs.

Case no.	Tube length (mm)	Side punch displacement (mm)
1	60	6
2	60	7
3	60	8
4	65	6
5	65	7
6	65	8
7	70	6
8	70	7
9	70	8

## Finite element analysis (FEA)

### Computer-aided design (CAD) model

**Fig 9:**
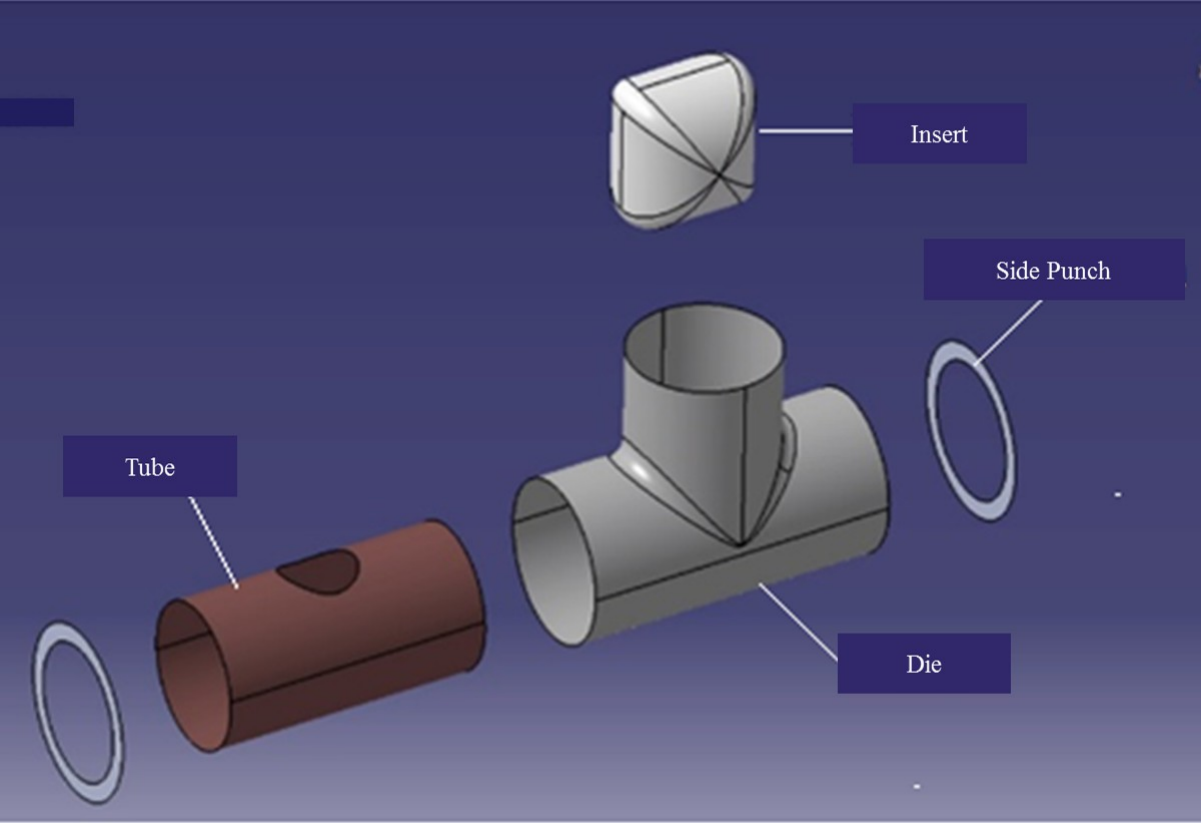
Computer-aided design (CAD) model.

A CAD model was built to simulate the manufacturing process of the T-shaped tube fittings using CATIA software. The model consists of five parts: (a) die, (b) tube, (c) right punch, (d) left punch, and (e) insert, as shown in Fig 9. All parts were modeled with two-dimensional (2D) elements (the element thickness was zero). The inner cavity of the die has a T-shape with horizontal length of 80mm, height of 35mm, and diameter of 35.05mm.

The FEA model followed the DOE outlined in the previous section with three trial lengths of the tube: 60, 65, and 70mm. The tube diameter and thickness were 35mm and 1.6mm, respectively. The insert was modeled as the geometric intersection of two equal perpendicular cylinders of 31.8mm diameter (inner diameter of tube). Two side punches with internal diameter 28mm and external diameter 38mm were modeled to simulate side displacements.

### Finite element model

**Fig 10:**
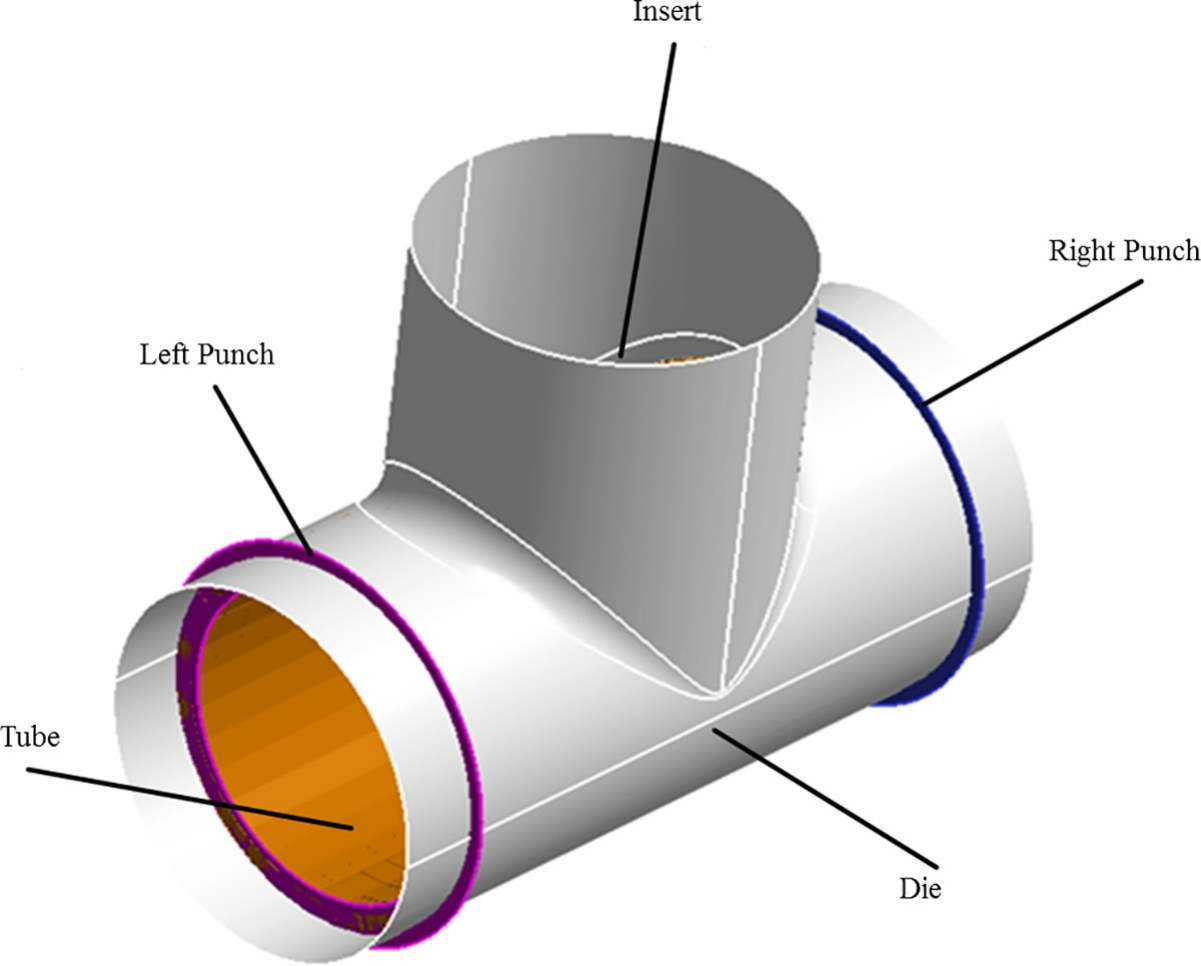
Finite element model.

A finite element model was created and solved using the DYNAFORM explicit pre-processor and LS-DYNA solver. Parts of the finite element model are shown in Fig 10. The tube was discretized to 1.6mm thick shell elements, and the number of elements and nodes were 638 and 1356, respectively. The die, insert, left punch, and right punch were discrete to rigid, where the number of elements and nodes in the die were 538 and 511, respectively, the number of elements and nodes in an insert were 592 and 542, respectively, and the number of elements and nodes in the left and right punches were 32 and 56, respectively. To eliminate the effects of mesh dependency, the model results were compared with a finer mesh and an optimum mesh size was selected based on accuracy and computational time. The interface contact between the tube, die, two punches, and insert was simulated using the surface-to-surface one-way forming algorithm that is defined in LS-DYNA (there is no contact between the die and the punches in the model). A friction coefficient of 0.18 was used to simulate the friction behavior between the contact surface of the tube and the die.

The LS-DYNA isotropic plasticity model is used, as given in Eq 1[18].

$$\sigma =k\varepsilon^{n},$$ Eq 1

where σ is the stress, ε is the strain, k is the strength coefficient, and n is the hardening exponent. The values of constants n and k are presented in Table 1.

## Measurements

**Fig 11:**
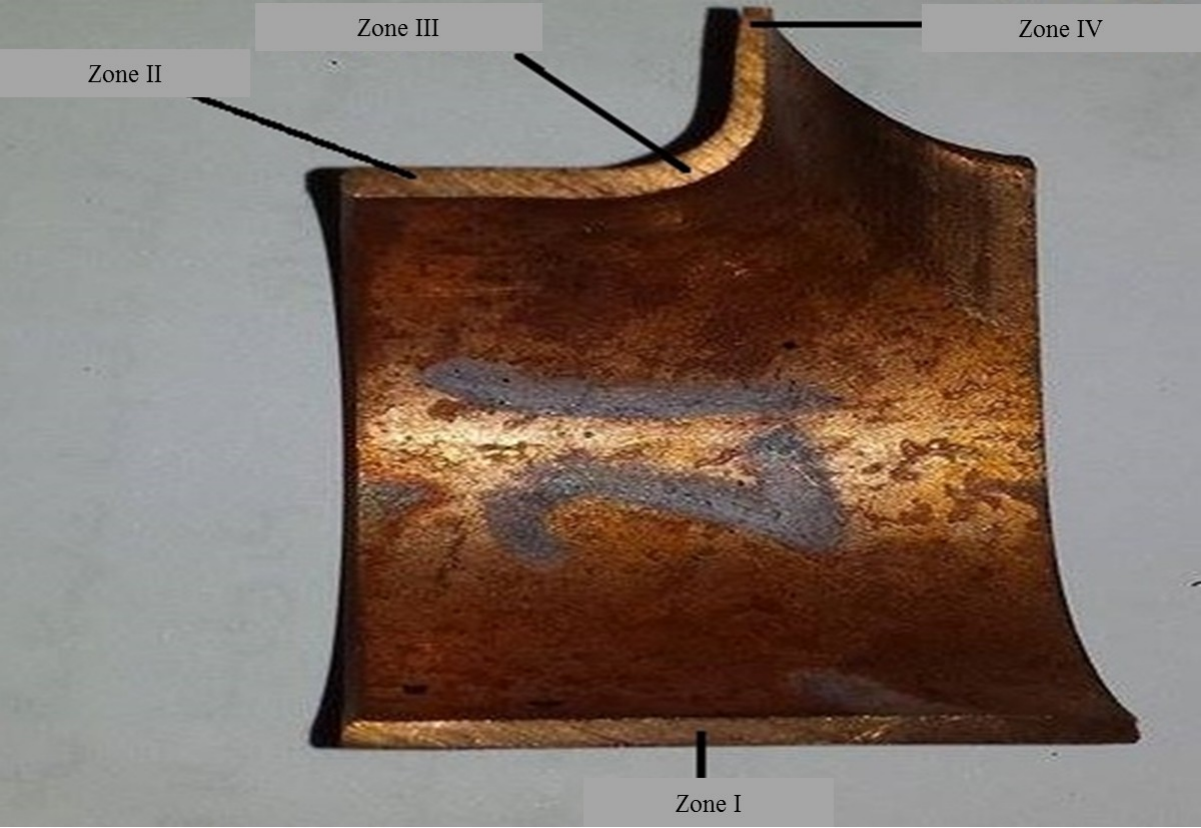
Cross-section of tube after experiment.

The phenomenon of thinning in T-shaped tube fitting is in contradiction with producing a sound and reliable product. To maintain product quality, thinning must be kept to minimum levels. After forming, the tube specimens were cut using an abrasive cutting machine. Fig 11 shows the cross-section of the tube after cutting. An INFINITY2 Lumenera microscope was used to measure the tube thickness distribution in four zones. A parameter defined as relative thickness (RT) was used for estimating the thinning and calculated as shown in Eq 2.

$$\mathrm{RT}=\frac{t}{t_{0}},$$ Eq 2

Where *t*_0_ is the initial thickness of the tube and *t* is the measured wall thickness after forming. Zones I-IV, where measurements took place, is shown in Fig 11.

## Results and discussion

### Experimental results

Reviewing the relative thickness measurements, zones I–III suffered from thickening with no existence of serious threats and with RT values ranging between 1.0 and 1.05. The thickening shows the flow of material from the sides of the tube workpiece toward its center due to the pressure of the side punches. Only zone IV at the top branch suffers thinning. Zone IV is always in contact with the moving insert during the shaping process and the flow of metal to this area is less than the flow of metal needed to produce the branch.

**Fig 12:**
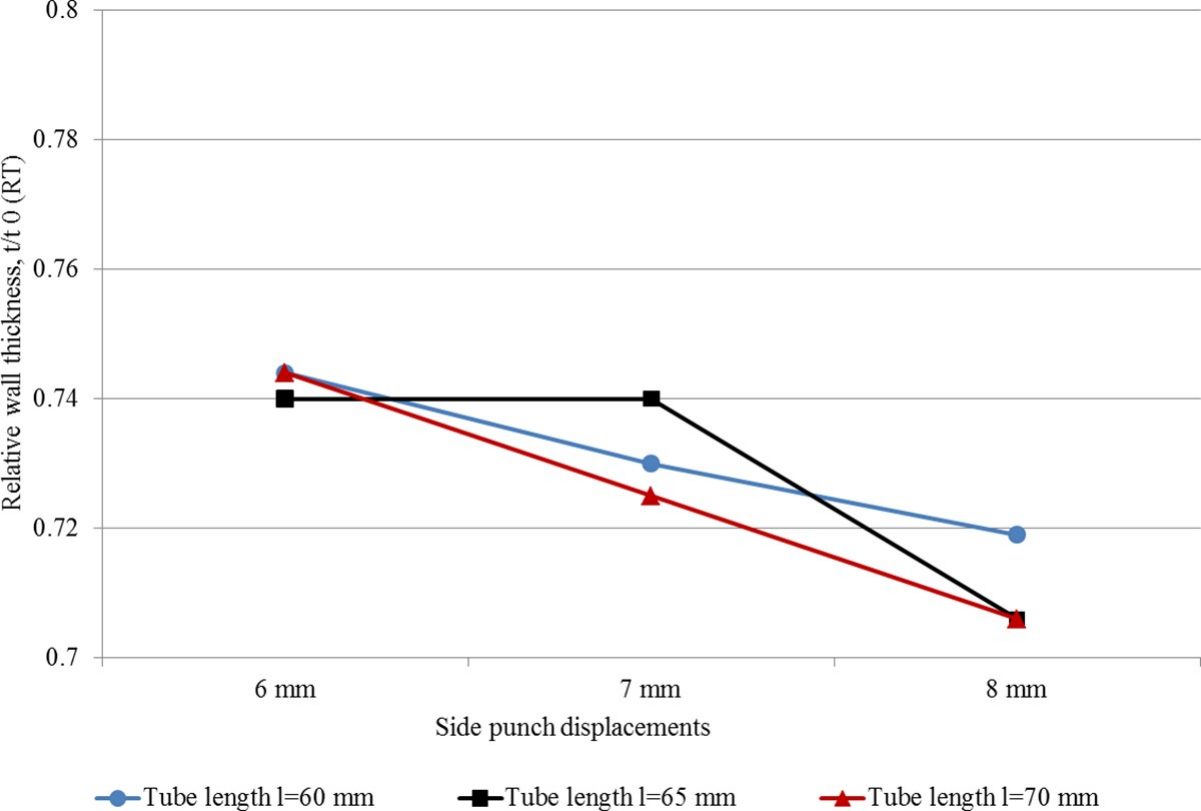
Experimental results of minimum RT.

Table 4 shows the experimental results for minimum RT (corresponds to maximum thinning) as an average for each case. It could be observed that the minimum RT ranges between 0.71 and 0.74. These values are better than those achieved by THF, even with the use of special setups [19]. Fig 12 shows the influence of process parameters (axial displacement and tube length) on RT. It could be observed that RT decreases with the increase of both SPD and TL. The highest branch height reached using the proposed new technique was 12.2 mm which is comparable to some research on THF output. For example, Alaswad et al. [22] who produced T-shaped tubes with a branch height of 11.6mm, while Mingtao et al. [19] produced T-shaped tubes with branch heights of more than 17mm using a special three-stage punch shape.

**Table 4:** Experimental results for minimum RT in zone IV.

Side punch displacement	Experimental minimum (RT) for zone IV		
	Tube length l = 60 mm	Tube length l = 65 mm	Tube length l = 70 mm
6 mm	0.74	0.74	0.74
7 mm	0.73	0.74	0.73
8 mm	0.72	0.71	0.71

### Simulation results

#### Thickness distribution

**Fig 13:**
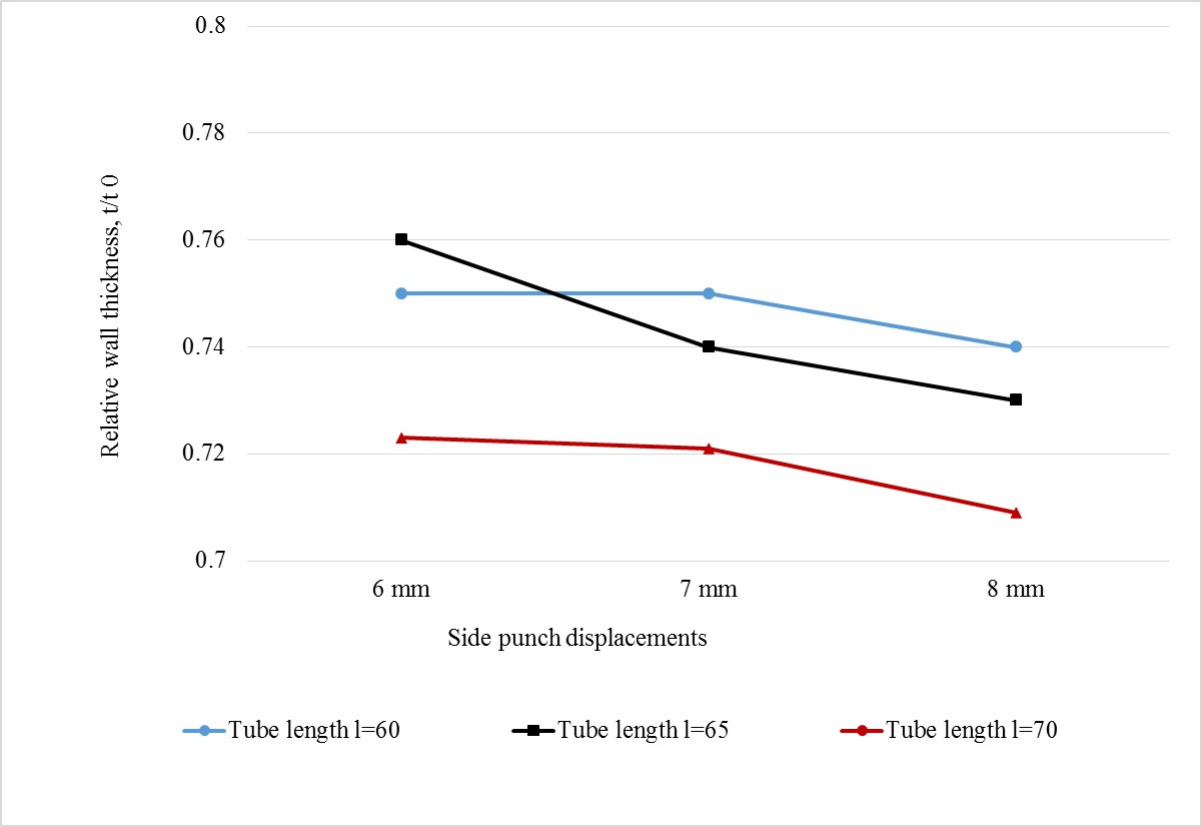
Simulation results of minimum RT.

**Fig 14:**
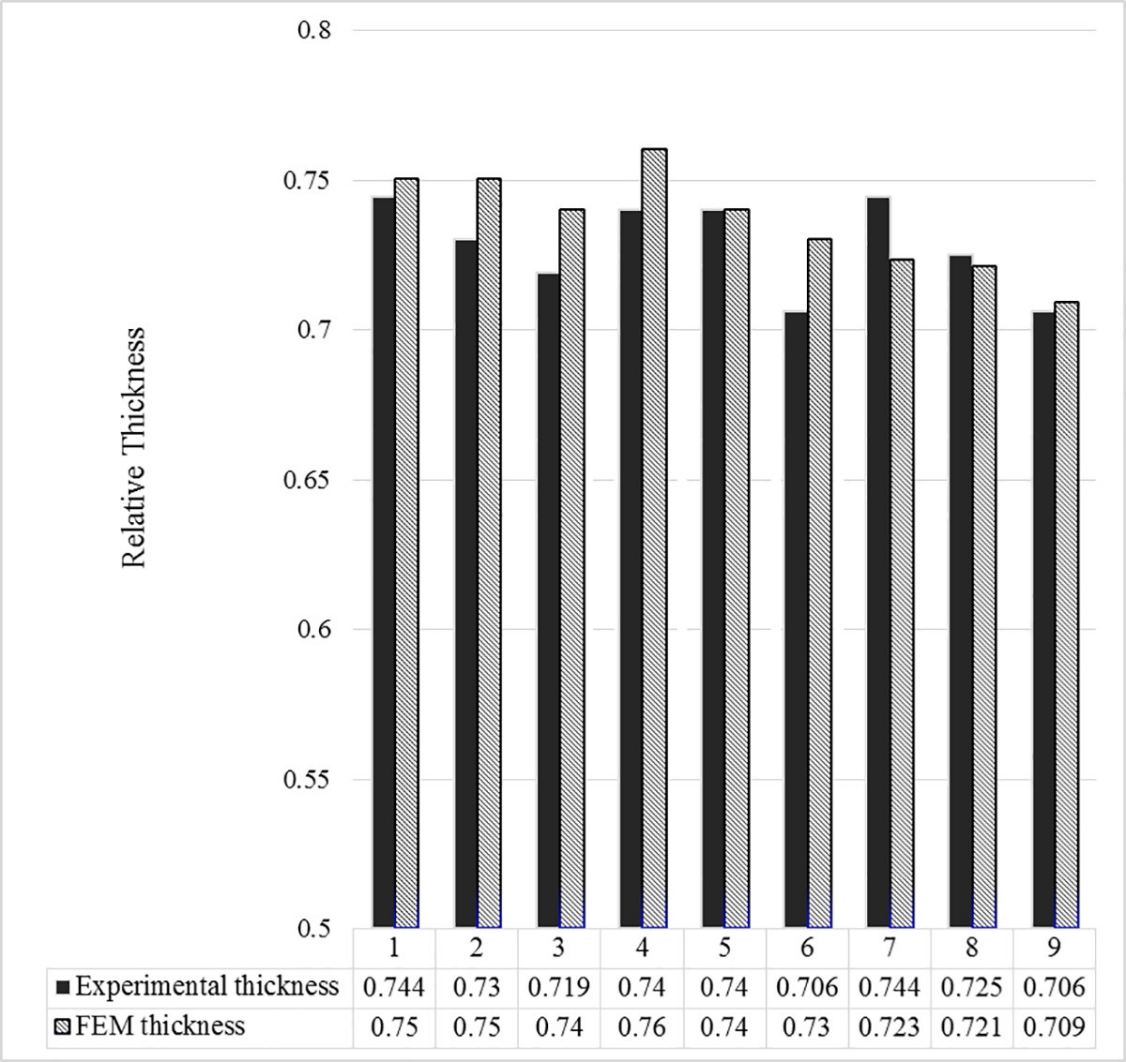
Experiments and finite element analysis (FEA) results.

Table 5 summarizes the RT values predicted by the FEA simulation. It can be seen that the predicted RTs match those measured experimentally, both in value and trend. The minimum RT, which corresponds to maximum thinning of 0.71, occurs in long tube workpieces and large side displacement. Fig 13 shows the RT change pattern with both factors. The column chart illustrated in Fig 14 proves the very close match between experimental and simulation results.

**Table 5:** Simulation results for minimum RT in zone IV.

Side punch displacement	Simulation results for RT for zone IV as averages		
	Tube length l = 60 mm	Tube length l = 65 mm	Tube length l = 70 mm
6 mm	0.75	0.76	0.72
7 mm	0.75	0.74	0.72
8 mm	0.74	0.73	0.71

**Fig 15:**
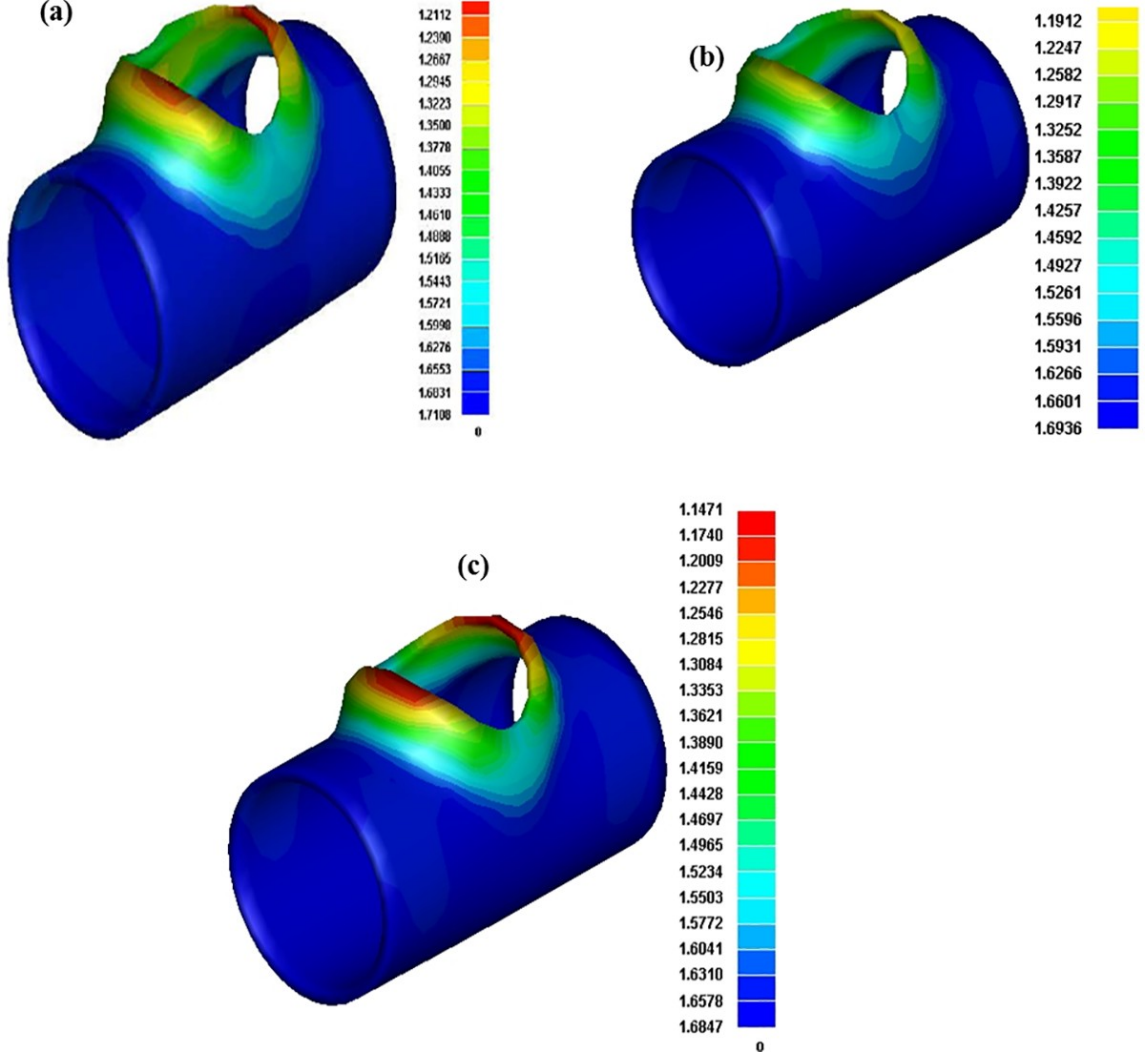
Distribution of tube thickness from FEA simulation a) Case 1 b) Case 4 c) Case 7.

Fig 15 presents snapshots of contours for thickness variation in different cases predicted by FEA. It can be observed that an increase in thickness is predicted in zones I, II, and III, while a decrease is predicted in zone IV, which is the same as experimental measurements. In all cases the maximum thinning occurs at the top of the branch.

#### Stress-strain state

**Fig 16:**
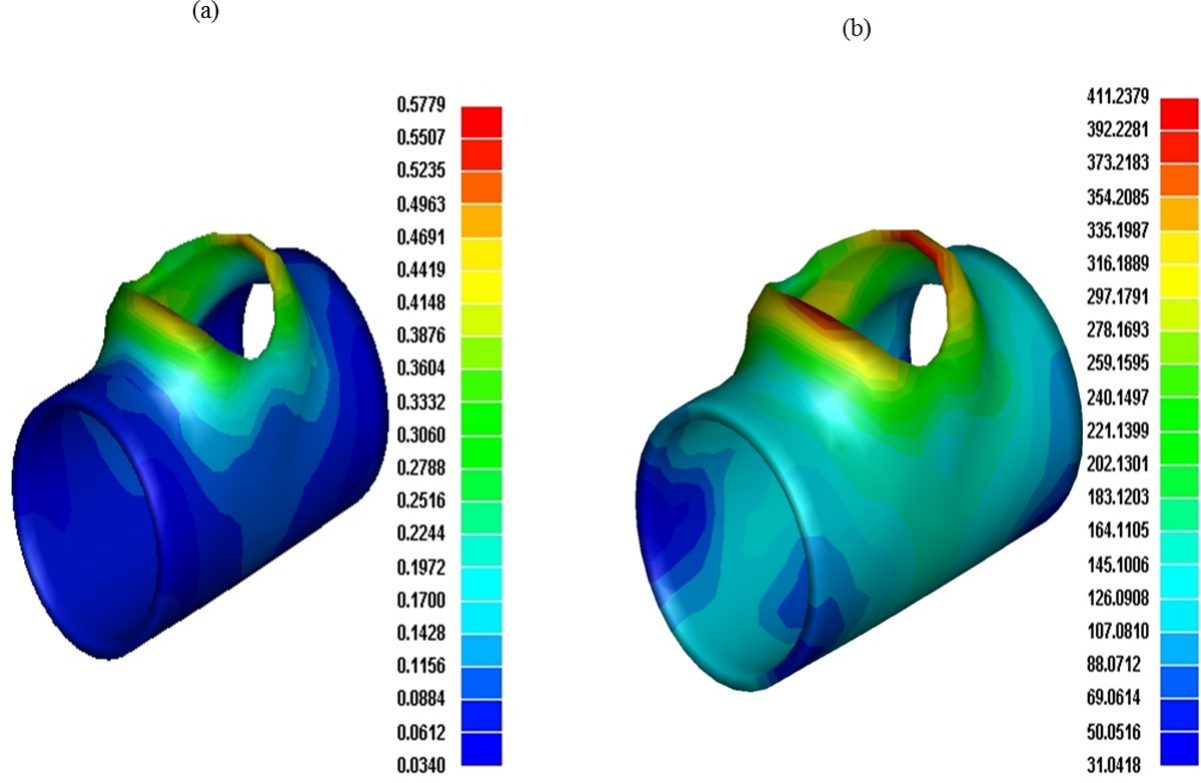
Effective strain (a) and effective stress (b) in Case 1.

Fig 16 shows the effective plastic strain and effective stress (in MPa) at the end of the forming process in Case 1. It is observed that the maximum effective plastic strain and maximum effective stress are concentrated at the highest point of branch, as expected. The maximum effective strain was 0.58 while the maximum effective stress was 411 MPa. These values are comparable to the results obtained by Mingtao et al. [19] and Crapps et al. [20] in hydroforming using annealed copper tube of almost the same size.

While the maximum stresses and strains are comparable to those of THF, the stress and strain distributions show better patterns using the current proposed method. Considering the effective strain, most of the tube body has strain values less than 0.1. In the work of Mingtao et al. [19], using traditional punches, most of the strains are between 0.12 and 0.38. The effective stress is between 90 and 160 MPa, while in Mingtao et al., the stress was much higher.

### DOE results

Full factorial design with two factors (TL and SPD), three levels of each factor, and three replicates for each experiment, totaling 27 runs, was used in this research. Table 6 summarizes the minimum RT measured from the 27 runs. Analysis of variance (ANOVA) was used to estimate the effects of both factors and their interaction on the RT at zone IV, as outlined in section 7.1. As a standard practice with ANOVA; terms with p-value < α = 0.05 are considered significant.

**Table 6:** Minimum RT values measured from the 27 runs.

Case no.	Run1	Run2	Run3
1	0.743	0.742	0.747
2	0.727	0.735	0.732
3	0.725	0.713	0.718
4	0.741	0.743	0.742
5	0.731	0.741	0.733
6	0.708	0.702	0.709
7	0.742	0.743	0.746
8	0.723	0.725	0.728
9	0.71	0.702	0.707

Table 7 presents the ANOVA results for measured RT. The p-values for both factors and the interaction between them are less than 0.05, proving that the three terms have a significant effect on RT. The value of Adj.SS of a term (factor or interaction) is defined as the variation of the response explained by this term while the total Adj. SS represents the total variation of the response. The value of each term with respect to the total Adj.SS represents the contribution of this term to the total variation within the measured data. The numbers indicate that TL is the dominant term with 83% contribution while SPD and the interaction contributions are 2.8% and 9%, respectively.

**Table 7:** ANOVA results for minimum RT.

Source	DF	Adj SS	Adj MS	F-Value	p-Value
Model	8	0.00604	0.000755	45.2	0
Linear	4	0.00546	0.001367	81.82	0
TL	2	0.00529	0.002644	158.29	0
SPD	2	0.00018	0.000089	5.36	0.015
2-Way Interactions	4	0.00057	0.000143	8.58	0
TL*SPD	4	0.00057	0.000143	8.58	0
Error	18	0.00030	0.000017		
Total	26	0.00634			
Model summary	S 0.0040868 R-sq 95.26% R-sq(adj) 93.15%				

The model adequacy is measured through the values of the coefficient of determination (R^2^, adjusted R^2^, and predicted R^2^). In such a multi-factor model, adjusted R^2^ provides better representation than R^2^. A 95% value for adjusted R^2^ proves the model is adequate and explains 95% of the variation in the data. A 93% value for predicted R^2^ indicates that the model has a good predictability and is not over-fit.

**Fig 17:**
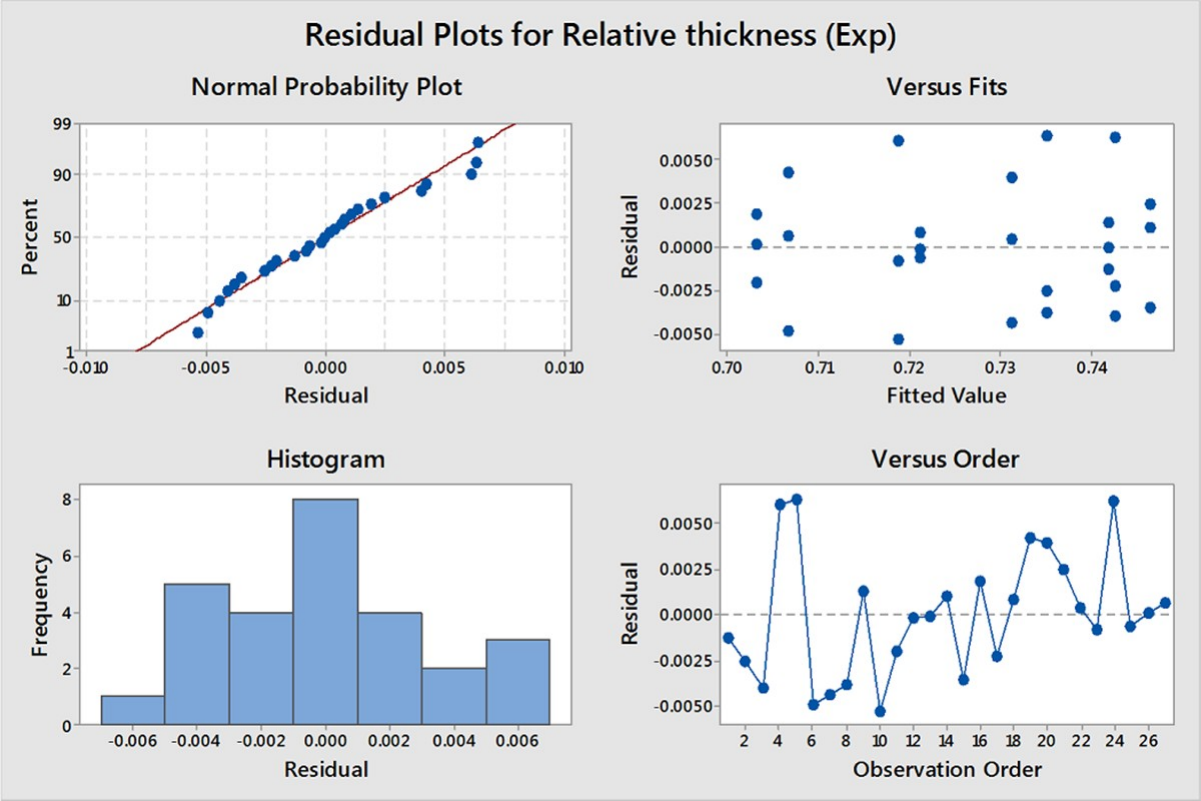
Residual plots RT.

**Fig 18:**
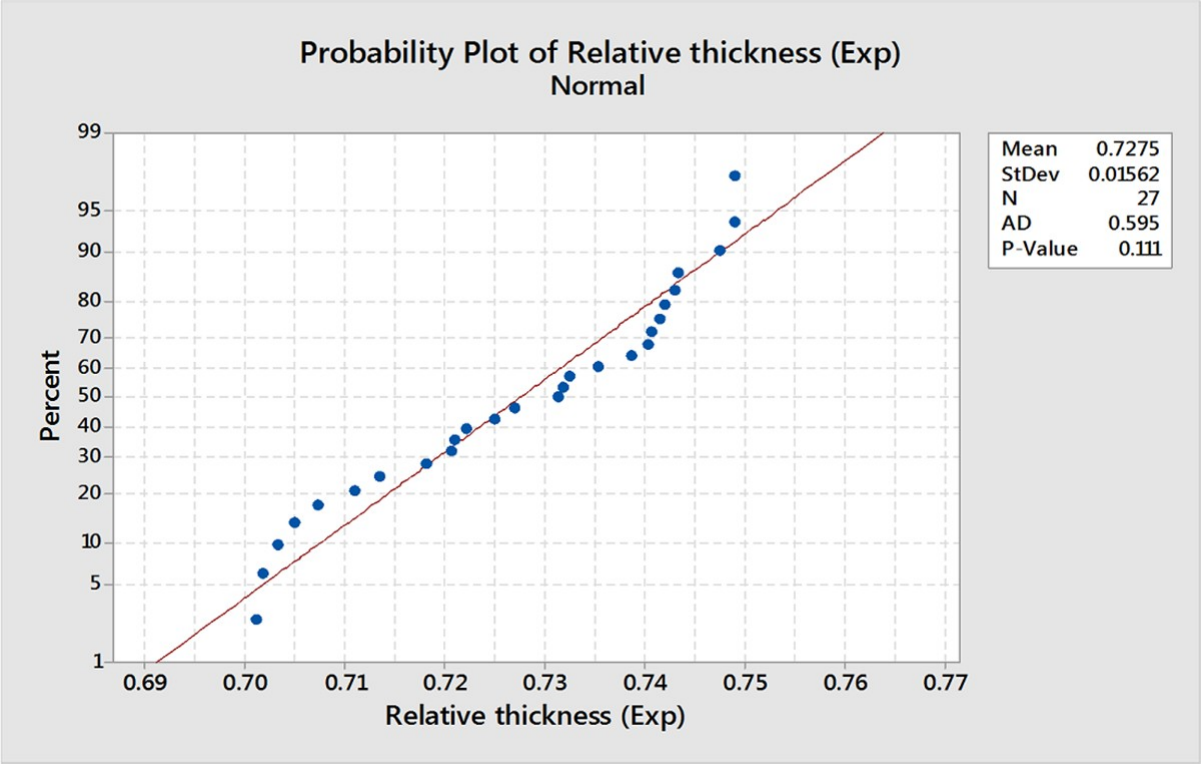
Normality test plot for RT.

Fig 17 shows a plot of the residuals versus fits and observation order. The residuals do not appear to show a pattern and are distributed randomly against both fitted value and observation order proving the validity of the assumptions of equal variance and independence, respectively. The Anderson-Darling test of the residual values resulted in p-value = 0.111 > 0.05, which proves that the residuals are normally distributed, as shown in Fig 18.

**Fig 19:**
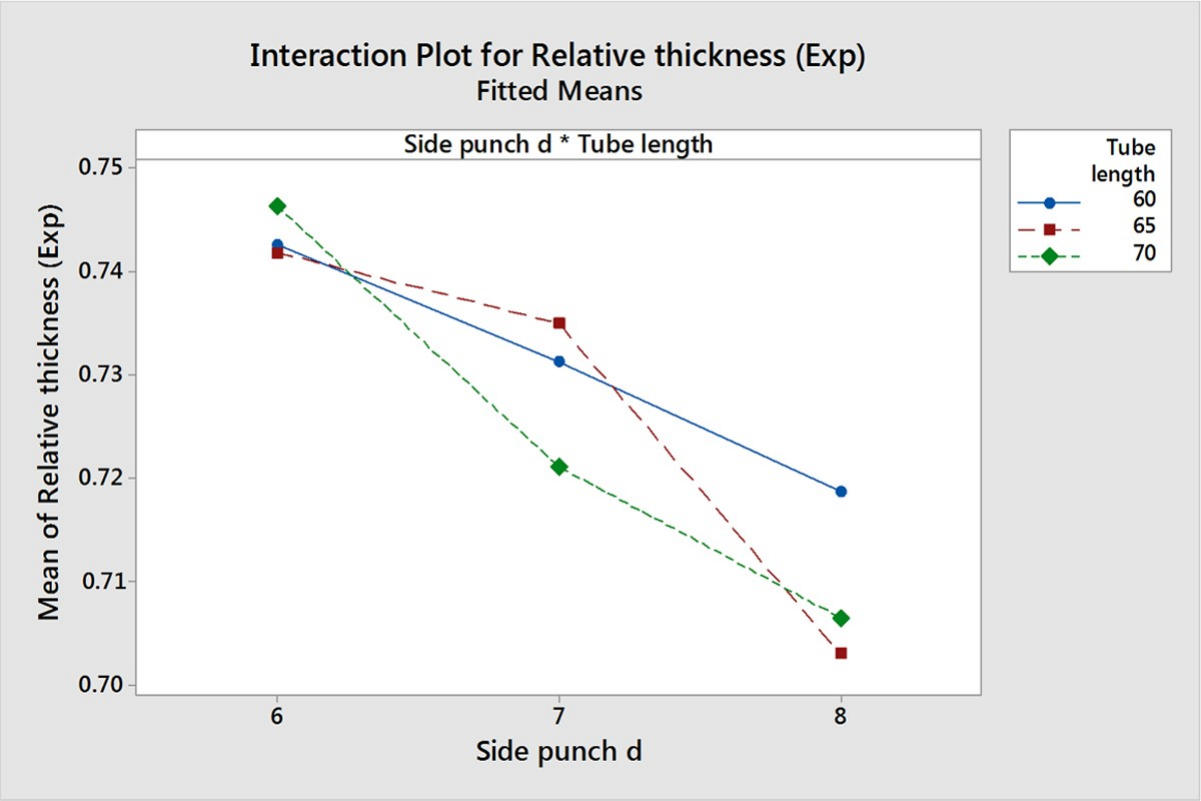
Interaction plot for RT.

Fig 19 illustrates the effects of interaction between TL and SPD on RT. The plot shows that high RT occurs at low SPD regardless of the TL value. When SPD is high, the TL value makes a difference as the low value of 60mm results in the highest RT.

## Conclusions

An innovative method to produce T-shape tube fittings was designed and successfully tested. The new technique resembles the hydroforming process while eliminating the need for internal pressure. Experimental and numerical analyses were used to assist the evaluation of the proposed manufacturing method. Thinning of produced parts was measured and compared to those resulting from FEA. A match between the experimental and numerical results was revealed.

Results indicate that the proposed technique produced parts comparable to THF in maximum thinning, branch height, effective strain, and effective stress. The proposed method resulted in better strain and stress distribution throughout the produced tube. The output of this study is limited to the same material and tube dimensions and cannot be extended beyond this without additional experimentation.
